# Return Strategy and Machine Learning Optimization of Tennis Sports Robot for Human Motion Recognition

**DOI:** 10.3389/fnbot.2022.857595

**Published:** 2022-04-28

**Authors:** Yuxuan Wang, Xiaoming Yang, Lili Wang, Zheng Hong, Wenjun Zou

**Affiliations:** ^1^Sports Institute, Nanchang JiaoTong Institute, Nanchang, China; ^2^Graduate School, University of Perpetual Help System Dalta, Las Piñas, Philippines; ^3^Faculty of Educational Studies, Universiti Putra Malaysia, Kuala Lumpur, Malaysia; ^4^College of Physical Education, East China University of Technology, Nanchang, China; ^5^School of Software, Nanchang University, Nanchang, China

**Keywords:** OpenPose traversal dataset, human motion characteristics, machine learning, support vector machine algorithm, human motion recognition

## Abstract

At present, there are many kinds of intelligent training equipment in tennis sports, but they all need human control. If a single tennis player uses the robot to return the ball, it will save some human resources. This study aims to improve the recognition rate of tennis sports robots in the return action and the return strategy. The human-oriented motion recognition of the tennis sports robot is taken as the starting point to recognize and analyze the return action of the tennis sports robot. The OpenPose traversal dataset is used to recognize and extract human motion features of tennis sports robots under different classifications. According to the return characteristics of the tennis sports robot, the method of tennis return strategy based on the support vector machine (SVM) is established, and the SVM algorithm in machine learning is optimized. Finally, the return strategy of tennis sports robots under eight return actions is analyzed and studied. The results reveal that the tennis sports robot based on the SVM-Optimization (SVM-O) algorithm has the highest return recognition rate, and the average return recognition rate is 88.61%. The error rates of the backswing, forward swing, and volatilization are high in the return strategy of tennis sports robots. The preparation action, backswing, and volatilization can achieve more objective results in the analysis of the return strategy, which is more than 90%. With the increase of iteration times, the effect of the model simulation experiment based on SVM-O is the best. It suggests that the algorithm proposed has a reliable accuracy of the return strategy of tennis sports robots, which meets the research requirements. Human motion recognition is integrated with the return motion of tennis sports robots. The application of the SVM-O algorithm to the return action recognition of tennis sports robots has good practicability in the return action recognition of tennis sports robot and solves the problem that the optimization algorithm cannot be applied to the real-time requirements. It has important research significance for the application of an optimized SVM algorithm in sports action recognition.

## Research Purpose and Current Situation

With the progress of computer technology and its wide application in life practice, behavior detection and action recognition projects based on various algorithms are being applied and studied in related fields (Aslan and Durdu, 2020). Behavior monitoring includes bee colony behavior and human behavior monitoring through graphics, temperature, humidity, sound, and other information (Jalal and Kamal, 2019). Introducing human motion recognition and motion intention prediction into the machine can bring the users a better sense of experience. People and robots coexist or work together. On the premise of ensuring that robots do not harm humans, it is necessary to explore efficient human–machine cooperation schemes to complement the advantages of humans and robots (Kahanowich, 2021). Generally, human motion is obtained in the form of a video or image and then recognized. With the development of science and technology and the electronic industry, wearable sensor devices are being used to recognize human actions (Hou et al., 2019; Jalal and Quaid, 2019; Zhao, 2020). With the growth of wireless technology and the expansion of coverage, wireless fidelity (WiFi) signal is being used to recognize human motion, and good results have been achieved, which is also the latest research trend (Zhao et al., 2019; Zhu et al., 2021). Generally, data are first collected, then denoised, and processed in human motion recognition. Then, feature quantity is extracted, trained, and classified to realize the final recognition. Almost all research teams achieve human motion recognition according to this general process. In these five parts, data denoising and feature extraction are the two key links (Gurbuz, 2019; Wang and Zhang, 2019; Xiong et al., 2020). Researchers are deeply improving and developing these two links to improve recognition accuracy.

The training law of tennis strategy and tactics is a difficult work faced by coaches and players. In the evaluation study of tennis serve and return practice, Krause et al. (2019) found that every hitting technique and step running position of the opponent could determine the return quality. Athletes also need to combine their own playing characteristics and styles in peacetime training to figure out every technical detail of the opponent to benefit from it (Krause et al., 2019). People hope to find a method that can be applied to all players to make them adopt strategies and tactics in a broader range and find weaknesses through analysis. Human motion recognition is a new human–computer interaction mode. It extracts and classifies human action features through computer vision technology, identifies human actions, obtains action information, and makes the machine “read” human body language (Oudah and Al-Naji, 2020; Pourdarbani et al., 2020; Yang et al., 2021). At present, significant progress has been made in the research and application of machine learning represented by deep learning (DL), which has effectively promoted the development of artificial intelligence. DL is a method of machine learning. In 2010, a large image dataset named ImageNet appeared. DL requires a lot of computational power, so some researchers combine the central processing unit (CPU) to train the DL model, which has been integrated into the current research method. Multiple researchers do various experiments through the standard datasets of machine learning and promote the research process by comparing the different methods. Giles and Kovalchik (2020) studied the direction change in the tracking data of professional tennis players and found that the support vector machine (SVM) algorithm tested the non-linear kernel method. The nearest neighbor method was used to test the simple neural network. It was usually recommended to experiment with the data of some shapes (Giles and Kovalchik, 2020; Kunze et al., 2020). ImageNet is one of the most influential datasets, which effectively promotes the development of the DL model (Alexopoulos and Nikolakis, 2020). Moreover, according to the tennis sports robots' return action selection and return strategy characteristics, there are many other datasets besides the ImageNet dataset. Based on that, many researchers also put forward new questions. These datasets also promote the development of relevant research (Liu et al., 2019).

For the problem of reduced human resources in tennis training, the robot can return the ball according to human actions. With the human motion recognition in the return strategy of tennis sports robots as the main starting point, this study extracts the human motion features through the tennis sports information under different classifications and the OpenPose traversal dataset. The return strategy of tennis sports robots is studied through machine learning. Besides, this study also focuses on the SVM algorithm optimization in machine learning. The return strategy of the tennis sports robot is optimized by the SVM-optimization (SVM-O) algorithm. This study provides a reference basis for robot return strategy in tennis sports and has crucial research significance.

Section Research Purpose and Current Situation introduces the research background of the machine learning algorithm of tennis robots for human motion recognition, which paves the way for the development of the SVM algorithm. Section Literature Review discusses the worldwide research on ball return strategy and human motion recognition. Section Research on Machine Learning and Related Sports Robots introduces machine learning and robots on the playground. Section Design of the SVM-O Model introduces the SVM algorithm and the optimization process of SVM. Section Research Model and Framework mainly aims at the ball return action recognition and ball return strategy design of the tennis robot. Section Results and Discussion analyzes the regression error rate, regression accuracy rate, regression recall rate, regression score rate, and regression recognition rate of eight actions in the tennis robot regression strategy and compares the regression accuracy rate of tennis robot under different model training. Finally, the whole research is summarized and analyzed, and the research limitations are put forward.

## Literature Review

According to the research on the return strategy, Sharma and Kumar (2021) designed the badminton robot through the learning method of the database and obtained the movement track of badminton through the binocular camera. However, due to the fixed base and position of the robot, the receiving distance was short when returning the ball. Yunardi et al. (2021) designed an omnidirectional mobile tennis robot. Through the design of a robot manipulator, the robot could return the ball at different angles and forces and could move in any direction and return the ball at different angles on the playground. Based on the fully connected neural network, the return strategy of the badminton robot was studied. The neural network was optimized through the activation function and residual connection of the neurons. The training was designed according to the return point of badminton as the input value. However, only the coordinates of the return point of the badminton in returning the ball were researched. The return type and the return action of the opponent were not taken into account, so it was difficult to return the ball for the action of the robot facing the human body (Gao et al., 2020). In the data-processing stage, three-dimensional (3D) is much more complex than two-dimensional (2D). The 2D human pose recognition is more mature than 3D in terms of data and models. The 2D models also have a lot of outdoor and natural data, but almost all 3D data have indoor data. Because of the complexity of the 3D annotation and recognition, massive sensors and cameras are needed to collect data. Before 2015, the regression method was widely used to confirm the coordinates of human-joint points. Its innovation was to extract them from the 3 and 7 layers of convolutional neural network (CNN) and then conduct the convolution operation, which is called the spatial fusion model. Faisal et al. (2019) used the spatial fusion model to extract the internal relationship between joint points, but this method was difficult to expand in the model. The critical path method (CPM) proposed in 2016 has strong robustness, and many subsequent methods are improvements based on this method.

The contribution of the CPM is to use sequential convolution architecture to express spatial and texture information. The network is divided into several stages, and each stage has a part in supervision and training. The previous stage uses the original image as the input. The latter uses the feature image of the previous stage as the input, mainly to integrate spatial information, texture information, and center constraints. In addition, Li et al. (2020) simultaneously used multiple scales to process the input characteristics and responses for the same convolution architecture, ensuring accuracy and considering the distance relationship between the various components. Fine-tuning was conducted based on CNN. Its innovation lies in the use of a geometric transformation kernel in the convolution layer, which can model the dependence between the joint points. Moreover, a bidirectional tree model was proposed, so that the feature channel of each joint could receive the information of the other joints, which was called information transmission. This tree structure can also estimate the attitude of multiple people. However, the accuracy of this multi-person attitude estimation is not high, and the method based on a single person is better. OpenPose is a framework for real-time estimation of the human body, face, and hand morphology proposed by the cognitive computing laboratory of Carnegie Mellon University (Jaruenpunyasak et al., 2022). It provides 2D and 3D multi-person keypoint detection and a calibration toolbox for estimating specific area parameters. It can accept many kinds of input, including images, videos, and webcams. Similarly, the output of OpenPose is also diverse. The input and output parameters can also be adjusted according to different needs. At present, the sensor technology of human motion recognition takes the human motion posture database as the action classifier, obtains the image of human motion through the sensor, subdivides it according to the motion angle and speed, and finally classifies the information with the classifier.

This thesis uses the OpenPose traversal dataset to extract the information features of human motion recognition nodes and extracts human motion features through tennis motion information under different classifications and the OpenPose traversal dataset. The return strategy of the tennis robot is studied by machine learning and optimized by the SVM-O algorithm.

## Research on Machine Learning and Related Sports Robots

### Algorithm Design of Machine Learning

**Figure 1:**
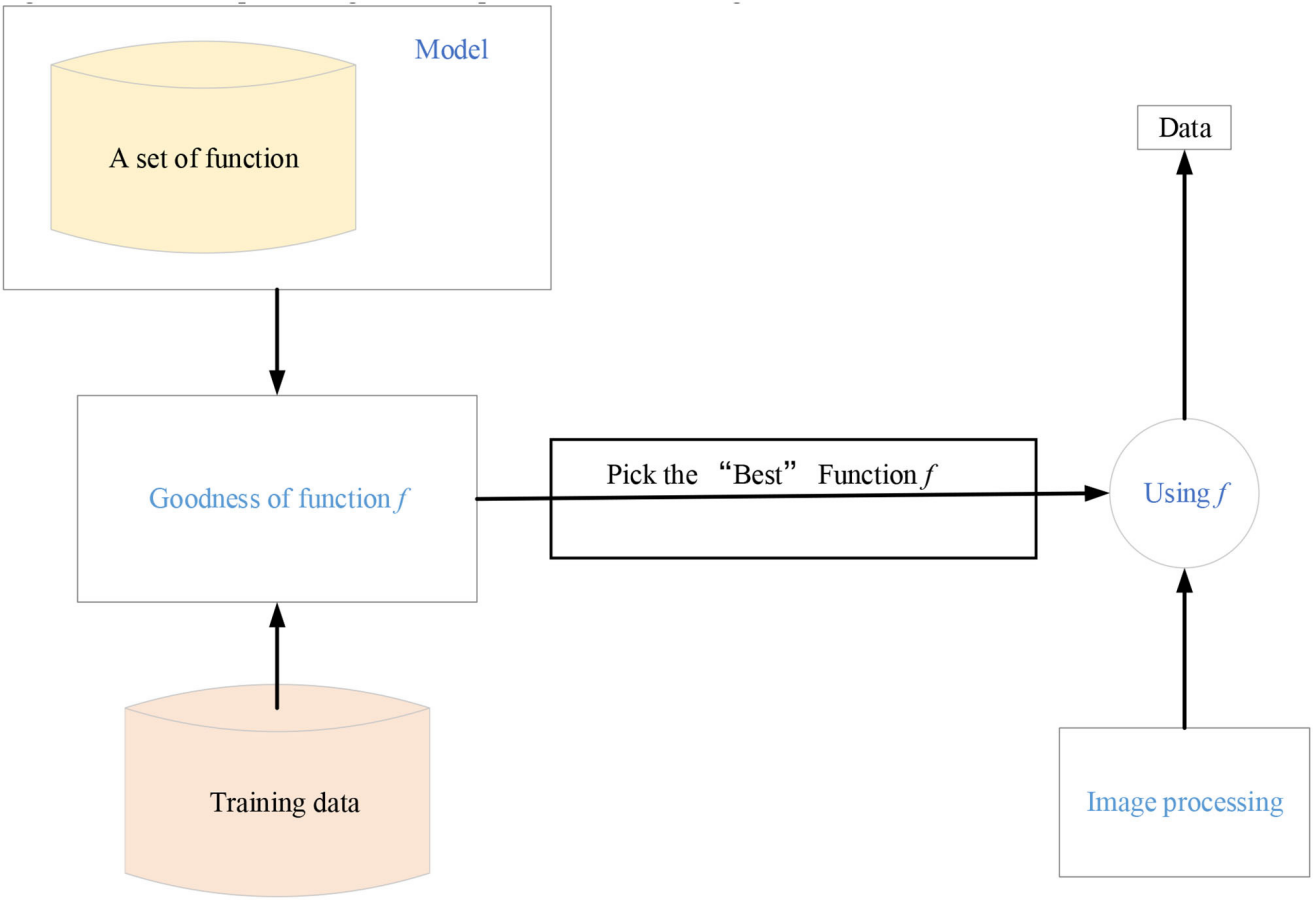
Machine learning steps.

Machine learning is the general name of a class of algorithms. These algorithms intend to mine the hidden laws from massive historical data and use them for prediction or classification. More specifically, machine learning can be seen as looking for a function, whose input is the sample data and the output is the expected result. However, this function is too complex to be expressed formally (Mohabatkar and Ebrahimi, 2021). It should be noted that the goal of machine learning is to make the learned functions well applicable to the new sample data, not just perform well on the training samples (Ghorbanzadeh et al., 2019). The ability of the learned function to apply to new samples is called generalization ability. Figure 1 shows the specific algorithm steps of machine learning.

**Figure 2:**
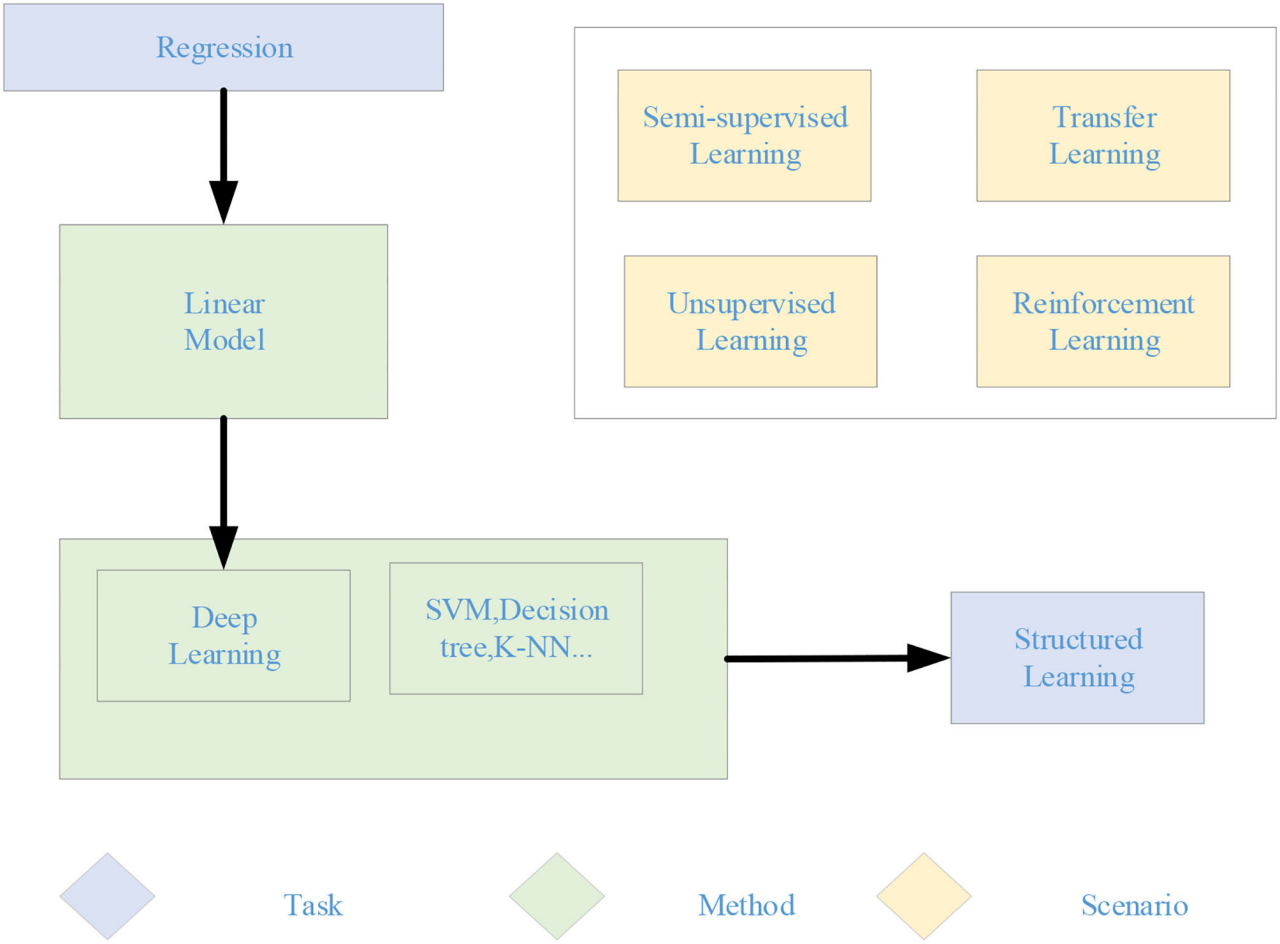
Roadmap for machine learning.

The machine learning steps in Figure 1 are divided into the following three steps. First, an appropriate model is selected, which usually depends on the actual problem. Suitable models need to be selected for different problems and tasks. The model is a set of functions. Next, the quality of a function is judged, which needs to determine a measurement standard, that is, the loss function. The determination of loss function also depends on specific problems. For example, Euclidean distance is generally used in regression problems, and the cross-entropy cost function is generally used in classification problems. Finally, the “best” function is found. The commonly used methods include gradient descent algorithm, ordinary least squares, and other tricks. The “best” function needs to be tested on a new sample after it is learned. It is a good function only if it performs well on the new sample. Machine learning is a huge family system, involving multiple algorithms, tasks, and learning theories. Figure 2 is the learning roadmap of machine learning.

In Figure 2, different colors represent different learning theories—orange represents tasks and green represents methods. According to the task types, machine learning models can be divided into regression, classification, and structure. The regression model is also called the prediction model. The output is a numerical value that cannot be enumerated. The classification models are divided into binary classification and multi-classification. The common binary classification problems are spam filtering, and the common multi-classification problems are automatic document classification. The output of the structured learning model is no longer a value with the fixed length, but the text description of the figure. From the perspective of the method, it can be divided into linear and non-linear models. The linear model is relatively simple, but its role cannot be ignored. It is the basis of the non-linear model. Many non-linear models are transformed from the linear model. The non-linear models can be divided into traditional machine learning models, such as SVM, K-nearest Neighbor (KNN), decision tree, and DL model. According to the learning theory, the machine learning model can be divided into supervised, semi-supervised, unsupervised, transfer, and reinforcement learning. When the training sample is labeled, it is supervised learning. When part of the training sample is labeled and part of the training sample is not labeled, it is semi-supervised learning. When all training samples are unlabeled, it is unsupervised learning. Transfer learning is to transfer the trained model parameters to the new model to help in the new model training. Reinforcement learning is to learn the optimal policy, which enables an agent to act according to the current state in a specific environment to obtain the maximum reward. The most significant difference between reinforcement learning and supervised learning is that each decision in reinforcement learning does not define right or wrong, but aims to get the most cumulative rewards.

### Robots on the Playground

Robot technology became the hottest topic in the investment circle together with unmanned aerial vehicle technology as early as 5 years ago. However, this topic was not pushed to the sports world until the “human–machine war” between Google AlphaGo and South Korean chess player Li Shishi.

The Japanese Volleyball Association uses robots to practice with athletes to improve the level of the athletes. This “defense robot” has three pairs of mechanical arms, which can move from one end to the other in front of the net to imitate the action of defense when smashing the attacker. The defensive robot is studied by the Japanese Volleyball Association and the University of Tsukuba. The coach can manipulate the robot arm back and forth according to the training needs. If the coach thinks the team can perform better in a past game, the robot can reproduce the game's situation at that time. The robot can imitate the possible actions of the opponent on the field in the future according to the strategic style of the opponent in the future. After the training starts, the coach only needs to press a button, and the robot's mechanical arm will quickly reach the designated position. The moving speed of this robot can reach 3.7 m/s, which is faster than that of the ordinary athletes running on the field.

Robomintoner, developed by Chengdu Electric Technology Chuangpin Robot Technology Co., Ltd., has reached the level of ordinary badminton lovers. Its technical core is the positioning and navigation of the whole field, the visual tracking and recognition of high-speed moving objects, and the control of the motion system. The robot mainly recognizes the trajectory of the badminton through binocular vision and predicts the landing point. It will tell the motion system the landing point of the badminton through Bluetooth communication, and the robot will move to the position where the badminton will land in advance.

The track and field training robots have a speed of 44.6 km/h, which is comparable to the world record of Usain Bolt in 2009. The main function is to see the difference between the user's current performance and the previous performance in real-time by simulating the runner's previous best performance during training to help the runner improve the performance. In terms of configuration, this small remote-controlled machine car has nine infrared sensors, drives small wheels through an Arduino board, and is equipped with a light-emitting diode (LED) light and a GoPro camera on the body.

The table tennis serving robot users can control the five built-in motors of the equipment in the supporting application of Trainerbot and set the serving into the up rotation, down rotation, side rotation, and random modes. The Trainerbot table tennis robot is 32 cm high and weighs only 1.2 kg. It can accommodate up to 30 table tennis balls. It can serve one ball every 0.5 s at the fastest and every 3 s at the slowest. Moreover, the table tennis coach robot can be used to recognize the ball's movement. The camera can track the position of table tennis 80 times per s, including ball speed, rotation speed, rotation direction, and other data. The sensor can analyze the speed of the ball 1,000 times per s and predict where the ball will land. Forpheus can calculate the angle and point at which the racket should hit back, with an error of <5 cm. Of course, the goal of Forpheus is to help mankind improve the level of table tennis. Therefore, in addition to tracking the table tennis ball, the landing point of the table tennis ball will be displayed by projection to help the athletes adjust their movements.

The tennis collection robot itself is equipped with advanced sensors. It detects, locates, and collects tennis balls by using the onboard computer to analyze the collected information and a wide-angle camera. In addition to the configured sensors, it also has a “Tennibot site” installed on the tennis net post. The site is a camera used to track the robot's position, which is connected with the robot through wireless communication. With the site to detect the robot's position, the robot's supporting iPhone Operating System (iOS) and Android application (APP) can be used to control which area of the playground it drives, set specific routes, or manual control. It can even be operated on an Apple watch, which users can choose. It is to avoid the robot appearing at the foot and affecting the playing when the user plays. The speed of collecting the tennis balls is 1.4 miles/h, about 38 m/min. A basket can collect 80 tennis balls, with a battery life of 4–5 h and only 90 min of charging each time, which can meet the needs of long-term practice. After use, the robot can be lifted and transported away through the handle like a suitcase.

## Design of the SVM-O Model

Support vector machine is a binary classification model, which is initially used in the case of linear separability. According to the diversity of the human actions studied, more action samples can be classified reasonably and as quickly as possible. This thesis optimizes the SVM sample classification process.

**Figure 3:**
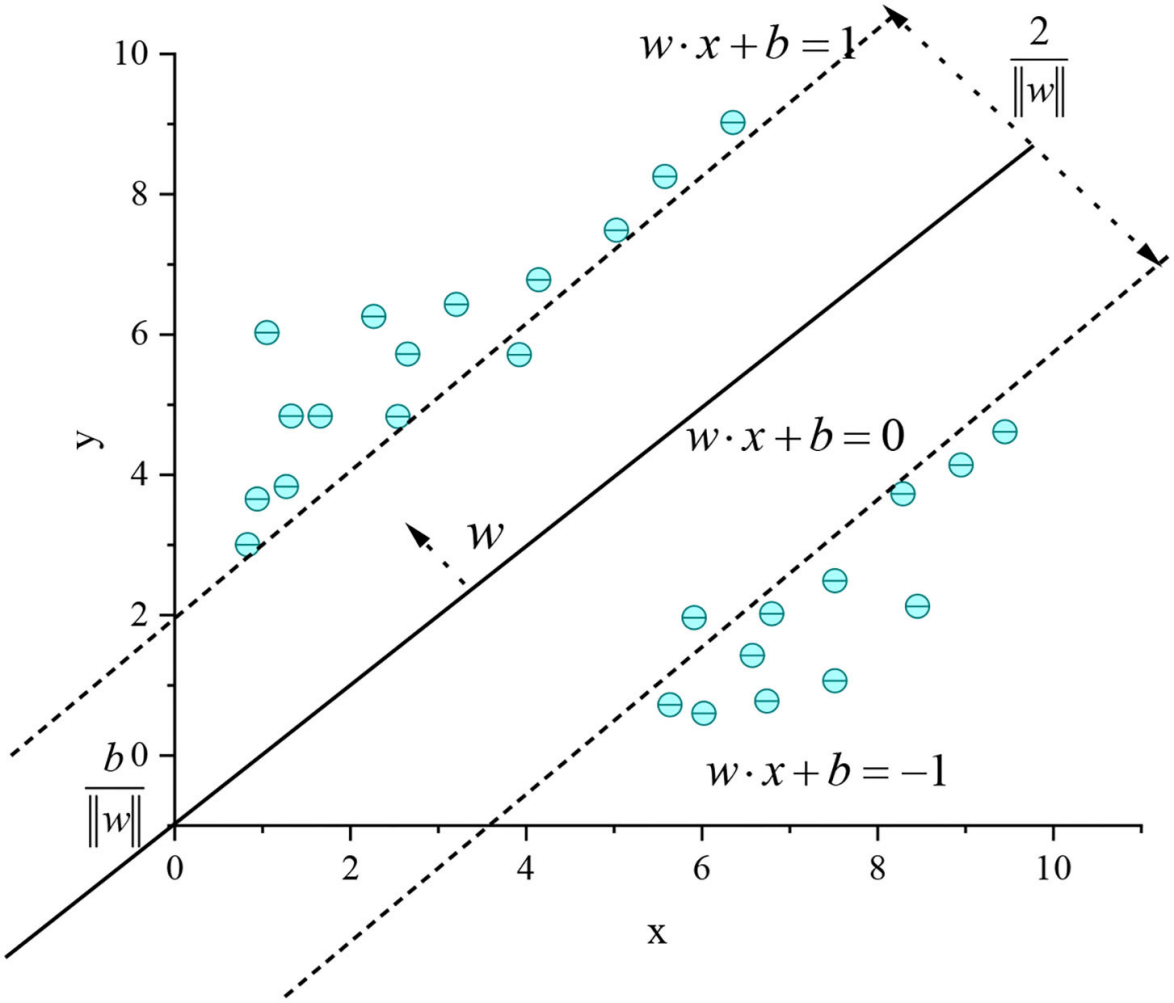
SVM regression model.

Given the training samples, the basic idea of the SVM algorithm classification learning is to find a partition hyperplane in the sample space based on the training samples to separate the samples of different categories. However, many partition hyperplanes can separate the training samples, and the most reliable one needs to be found (Shi, 2020). Intuitively, the one in the middle of the training sample should be found, because the partition hyperplane has the best tolerance to the local disturbance of the training sample. It means that the classification result of the partition hyperplane is the most robust and has the strongest generalization ability to the unseen examples. Figure 3 shows the SVM regression model.

In Figure 3, the hyperplane in the sample space can be divided by a linear equation *w* · *x* + *b* = 0, where *w* is the normal vector of the hyperplane partition, which determines the direction of the hyperplane; *b* is the displacement term, which determines the distance between the hyperplane and the origin. The partition hyperplane can be determined by *w* and *b*. The interval between two heterogeneous support vectors and the hyperplane is $\gamma =\frac{2}{\left\|w\right\|}$. In tennis sports, the robot return sample can be mapped from the original to a high-dimensional feature space, so that the sample can be linearly separable in this feature space. If the original space has a finite number of attributes, there must be a high-dimensional feature space to divide the return samples. ϕ(*x*) is set as the feature vector after *x* is mapped to the high dimension. Then, the model corresponding to ϕ(*x*) dividing the hyperplane in the feature space can be expressed as:


(1) $$\begin{array}{l} f\left(x\right)=w^{T}\phi \left(x\right)+b \end{array}$$

*w* and *b* are model parameters. There is


(2) $$\begin{array}{l} \min_{\left(w,b\right)}\frac{1}{2}{\left\|w\right\|}^{2},st,y_{i}\left(w^{T}\phi \left(x_{i}\right)+b\right)\geq 1,i=1,2,...,m \end{array}$$

Equation (3) is a dual problem.


(3) $$\begin{array}{l} \max_{\alpha }\left(\overset{m}{\underset{i=1}{\sum }}a_{i}-\frac{1}{2}\overset{m}{\underset{i=1}{\sum }}\overset{m}{\underset{j=1}{\sum }}\alpha_{i}\alpha_{j}y_{i}y_{j}\phi {\left(x_{i}\right)}^{T}\phi \left(x_{j}\right)\right)\\ s.t.\overset{m}{\underset{i=1}{\sum }}\alpha_{i}y_{i}=0,\alpha_{i}\geq 0,i=1,2,...,m \end{array}$$

$\phi {\left(x_{i}\right)}^{T}\phi \left(x_{j}\right)$ is the inner product after the training samples *x*_*i*_ and *x*_*j*_ are mapped to the feature space. The dimension of the feature space may be very high or even infinite. Therefore, the direct calculation is difficult, and a kernel function can be envisaged:


(4) $$\begin{array}{l} k\left(x_{i},x_{j}\right)\leq \phi \left(x_{i}\right),\phi \left(x_{j}\right)\geq \phi {\left(x_{i}\right)}^{T}\phi \left(x_{j}\right) \end{array}$$

Equation (4) is the inner product of *x*_*i*_ and *x*_*j*_ in feature space. It is equal to their calculation by function *k*(·, ·) in the original sample space. Equation (4) is solved to obtain the following:


(5) $$\begin{array}{l} f\left(x\right)=w^{T}\phi \left(x\right)+b=\overset{m}{\underset{i=1}{\sum }}\alpha_{i}y_{i}\phi {\left(x_{i}\right)}^{T}\phi \left(x\right)+b\\ =\overset{m}{\underset{i=1}{\sum }}\alpha_{i}y_{i}k\left(x,x_{j}\right)+b \end{array}$$

Common kernel function: Equation (6) is the Gaussian kernel function; σ is the bandwidth of the Gaussian kernel.


(6) $$\begin{array}{l} k\left(x_{i},x_{j}\right)=\exp\left(-\frac{\left\|x_{i}-x_{j}\right\|}{2\sigma^{2}}\right),\sigma >0 \end{array}$$

Equation (7) is the Laplace kernel function:


(7) $$\begin{array}{l} k\left(x_{i},x_{j}\right)=\exp\left(-\frac{\left\|x_{i}-x_{j}\right\|}{\sigma }\right),\sigma >0 \end{array}$$

Equation (8) is the Sigmoid kernel function; tanh is a hyperbolic tangent function.


(8) $$\begin{array}{l} k\left(x_{i},x_{j}\right)=\tanh\left(\beta x_{i}^{T}x_{j}+\theta \right),\beta >0,\theta <0 \end{array}$$

**Figure 4:**
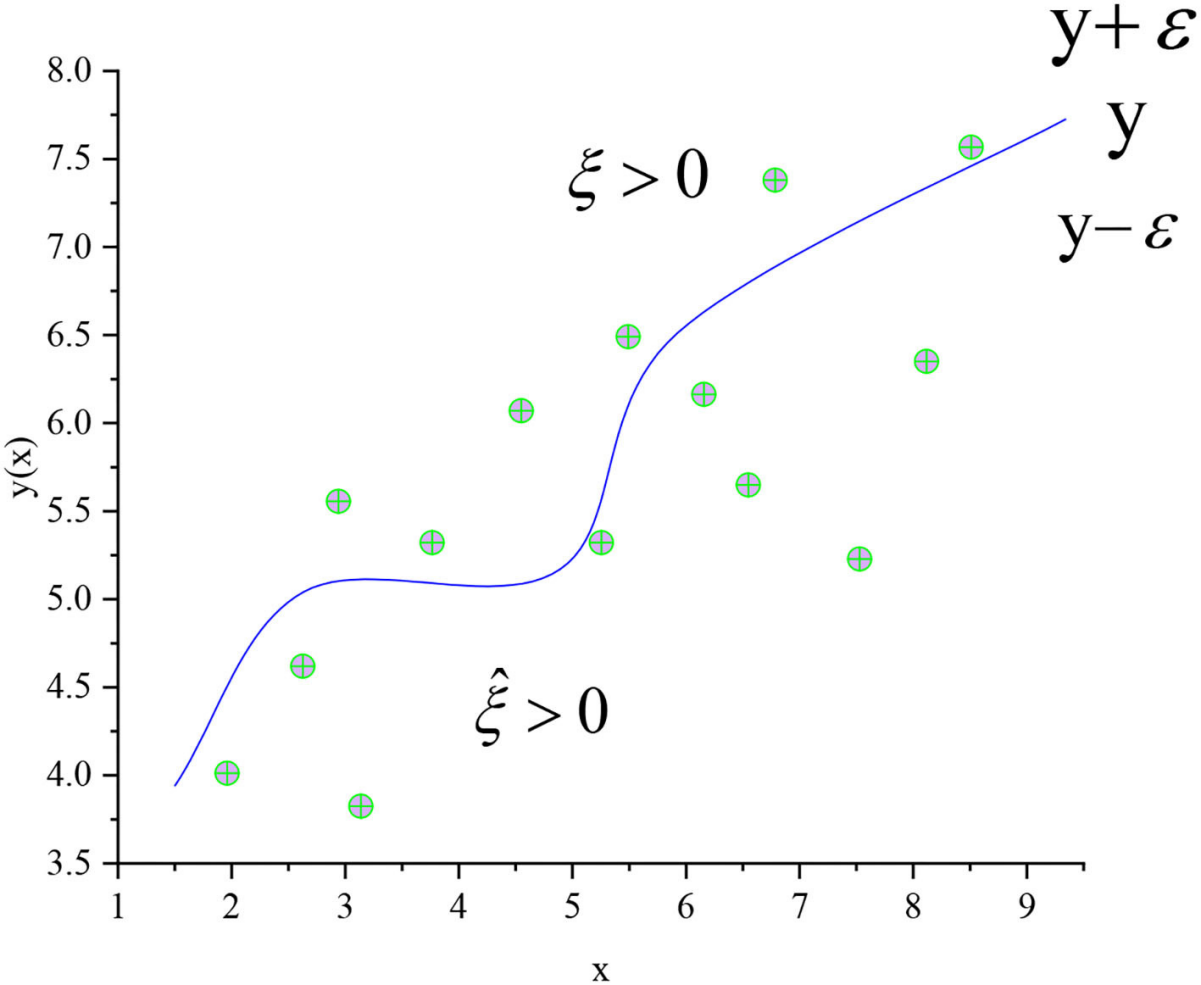
Regression curve of SVM-O.

All of the above samples must be divided correctly. The samples that do not meet the constraints can be optimized while maximizing the interval to reduce the error. The most commonly used error function is the least square sum error function. Figure 4 shows the optimization effect of the SVM regression curve.

In Figure 4, the regression curve of the SVM in Figure 3 is optimized. *C* is the regularization coefficient and the relaxation variable ξ is introduced. In the regression problem, each data point *x*_*n*_ needs two relaxation variables ξ_*n*_ ≥ 0 and ${\hat{\xi }}_{n}\geq 0$. ξ_*n*_ ≥ 0 corresponds to *t*_*n*_ > *y*(*x*_*n*_) + ε (above the curve in Figure 4) and corresponds to *t*_*n*_ < *y*(*x*_*n*_) + ε (below the curve in Figure 4). Equation (9) is a function that minimizes the regularization error. Equations (10) and (11) are the corresponding conditions.


(9) $$\begin{array}{l} C\overset{N}{\underset{n=1}{\sum }}E_{\varepsilon }\left(y\left(x\right)-t_{n}\right)+\frac{1}{2}{\left\|w\right\|}^{2} \end{array}$$

(10) $$\begin{array}{l} t_{n}\leq y\left(x_{n}\right)+\varepsilon +{\hat{\xi }}_{n}\\ t_{n}\geq y\left(x_{n}\right)-\varepsilon -{\hat{\xi }}_{n} \end{array}$$

The updated error function is as follows:


(11) $$\begin{array}{l} C\overset{N}{\underset{n=1}{\sum }}\left(\xi_{n}+{\hat{\xi }}_{n}\right)+\frac{1}{2}{\left\|w\right\|}^{2} \end{array}$$

The Lagrange multiplier method is introduced to optimize the equation with constraints and minimize the error function:


(12) $$\begin{array}{l} \begin{array}{c} L=C\overset{N}{\underset{n=1}{\sum }}\left(\xi_{n}+{\hat{\xi }}_{n}\right)+\frac{1}{2}{\left\|w\right\|}^{2}-\underset{n=1}{\sum }N\left(\mu_{n}\xi_{n}+{\hat{\mu }}_{n}{\hat{\xi }}_{n}\right)\\ -\overset{N}{\underset{n=1}{\sum }}a_{n}\left(\varepsilon +\xi_{n}+y_{n}-t_{n}\right)-\overset{N}{\underset{n=1}{\sum }}{â}_{n}\left(\varepsilon +{\hat{\xi }}_{n}-y_{n}+t_{n}\right) \end{array} \end{array}$$

By finding the derivative of the Lagrange function for *w, b*, ξ_*n*_ and ${\hat{\xi }}_{n}$ as 0, it can be obtained that


(13) $$\begin{array}{l} \begin{array}{c} \frac{\delta L}{\delta w}=0\Rightarrow \overset{N}{\underset{n=1}{\sum }}\left(a_{n}-{ã}_{n}\right)\phi \left(x_{n}\right)\\ \frac{\delta L}{\delta b}=0\Rightarrow \overset{N}{\underset{n=1}{\sum }}\left(a_{n}-{ã}_{n}\right)=0\\ \frac{\delta L}{\delta \xi_{n}}=0\Rightarrow a_{n}+\mu_{n}=C,\frac{\delta L}{\delta {\tilde{\xi }}_{n}}=0\Rightarrow {ã}_{n}+{\tilde{\mu }}_{n}=C \end{array} \end{array}$$

The new input variable can be obtained by using the equation below:


(14) $$\begin{array}{l} y\left(x\right)=\overset{N}{\underset{n=1}{\sum }}\left(a_{n}-{ã}_{n}\right)\phi \left(x_{n}\right)\phi \left(x\right)+b\\ =\overset{N}{\underset{n=1}{\sum }}\left(a_{n}-{ã}_{n}\right)k\left(x_{n},x\right)+b \end{array}$$

The value of *b* in Equation (1) can be obtained from Equation (15):


(15) $$\begin{array}{l} b=t_{n}-\varepsilon -w^{T}\phi \left(x_{n}\right)=t_{n}-\varepsilon -\overset{N}{\underset{m=1}{\sum }}\left(a_{m}-{ã}_{m}\right)k\left(x_{n},x_{m}\right) \end{array}$$

Another method for SVM regression does not fix the width of the insensitive area ε but fixes the proportion *v* of the data points outside the pipeline. The maximization equation can prove that at most *vN* data points fall outside the insensitive pipeline, and at least *vN* data points are support vectors on or outside the pipeline. The value of *vC* is generally determined by cross-validation.


(16) $$\begin{array}{l} \begin{array}{c} \tilde{L}\left(a,{ã}_{n}\right)=-\frac{1}{2}\overset{N}{\underset{n=1}{\sum }}\overset{N}{\underset{m=1}{\sum }}\left(a_{n}-{ã}_{n}\right)\left(a_{m},-{ã}_{m}\right)k\left(x_{n},x_{m}\right)+\overset{N}{\underset{n=1}{\sum }}\left(a_{n}-{ã}_{n}\right)t_{n}\\ 0\leq a_{n}\leq \frac{C}{N},0\leq {ã}_{n}\leq \frac{C}{N},\overset{N}{\underset{n=1}{\sum }}\left(a_{n}-{ã}_{n}\right)=0,\overset{N}{\underset{n=1}{\sum }}\left(a_{n}+{ã}_{n}\right)\leq vC \end{array} \end{array}$$

## Research Model and Framework

### Recognition of Return Motion of Tennis Sports Robot

The robot return in tennis sports cannot be realized only with a heavy swing. It needs to study the combination of human action routines and constantly return the ball to the other party. For example, in diagonal backhand, the robot needs to catch the opportunity ball that the opponent's oblique return angle is not good, the ball speed is not fast, and the ball falls in the center of the court or in a comfortable position in front of the body. The robot's backhand position can be changed to the forehand to play the diagonal ball and return the ball effectively. These return methods provide the structure of tennis return tactics and can be used to teach tennis sports robot return. Table 1 is the analysis of the return tactics of tennis sports robot.

**Table 1:** Analysis of tactical action characteristics of return ball of tennis sports robot.

**Return strategy**	**Characteristic**	**Optimization objectives**
Continuous return	Continuity	Change different ball speeds, forces and directions
Return with more strengths	Depth continuity, velocity	Return quickly
Moving return	Continuous power	Improve return accuracy and continuity
Return with endurance and defense	High endurance	Slash and straight ball
Changeable return	Change the speed, direction and kinetic energy of the ball	Optimize the rotation and direction of the shot
Attack front court	Aggressive	Return forehand, backhand

**Figure 5:**
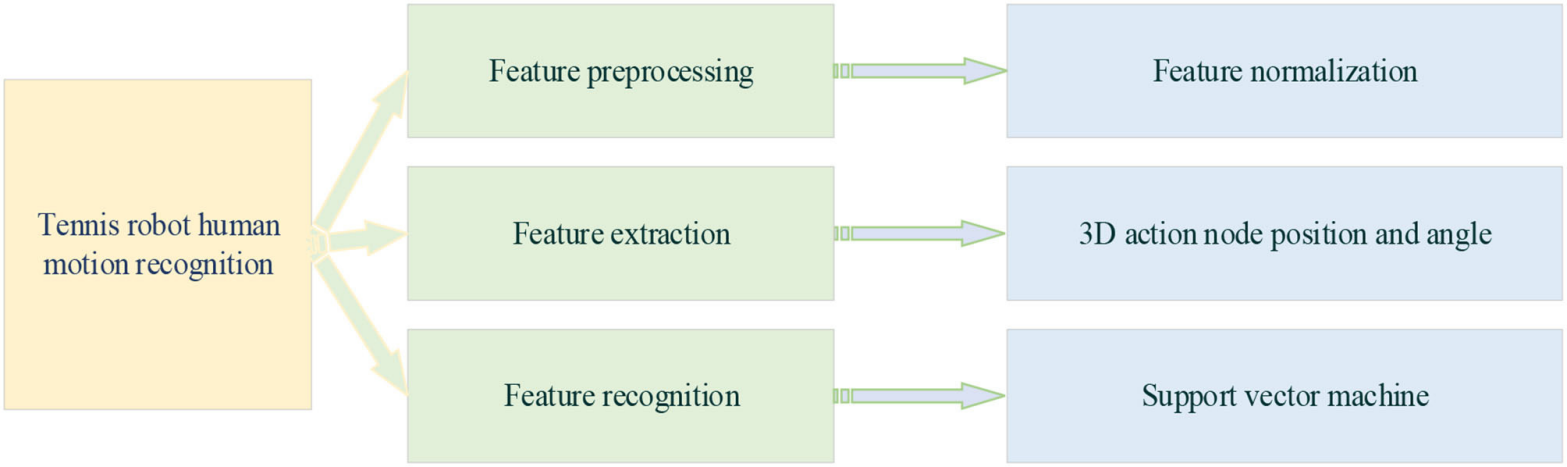
Motion recognition of tennis sports robots.

According to Table 1 and the high-performance camera processing of the tennis robot, the robot responds to the falling point of the opponent's returning the ball. The tennis movement is set as the return movement of 8 tennis sports robots, which are preparation action, swing, preparation position, backswing, forward swing, hitting, follow-up swing, and return to preparation position (Haryanto, 2020). In tennis robots' data-processing stage of return motion recognition, 3D human motion recognition is much more complex than 2D. Two-dimensional human motion recognition is more mature than 3D in terms of data and models. 2D models also have many outdoor and natural datasets. However, almost all 3D human motion recognition datasets are indoor. Because of the complexity of 3D annotation and recognition, many sensors and cameras are needed to collect data. Figure 5 is a motion recognition system of a tennis sports robot.

Figure 5 is the dataset feature extraction of the return strategy of tennis sports robots for human motion recognition. First, human motion recognition is carried out, followed by the extraction of motion features. The collected serving action images of the human body in tennis sports are put into the OpenPose skeleton extraction network to extract the keypoint coordinate data of human action. Then, the human action information under different classifications is extracted as action features and saved as the corresponding text (TXT) documents (Neff et al., 2019). Then, the features are integrated. The extracted feature information is integrated with the corresponding images in a TXT file, and the useless and redundant datasets are removed simultaneously. Finally, the TXT information is integrated as input and output tag comma-separated value (CSV) files, respectively. Among them, the input features include the key points of human service and the line features connected by different bone points and the surface features formed by the combination of different lines. These features will be extracted and learned through different classification algorithms.

In a convolutional neural network, the human motion figures are put into the network in the matrix form. The x, y, and z axes are taken as red, green, blue (RGB)—three channels of the figures. Then, the data are arranged in a row to get a vector with *n* columns. However, for convenience, *n* is decomposed. Every 100 groups of data form a return action figure of a tennis sports robot, that is, 1^*^100^*^3 represents a return action figure of a tennis sports robot. Of course, if the input is modified, the label file should also be modified accordingly. Before modification, each piece of data will have a label. However, after modification, every 100 pieces of data only need to output one label. Shell is adopted to process label files. Finally, the eight return motions in the label file are replaced with numbers from 1 to 8. The human bone information read from the input CSV is taken as the input and the CSV file of the *Y* label as the output. The training and verification sets are divided according to the proportion of 0.3. Then, they are converted into a NumPy matrix to participate in the operation. The decision tree, random forest, neural network, and SVM are selected as the machine learning model classifiers to test the model effect. The evaluation of the model is mainly based on the confusion matrix, accuracy, recall, and *f*_1_ score. Table 2 shows the recognition and evaluation results of the return motion of the tennis sports robot under different classifiers.

**Table 2:** Recognition and evaluation results of the return motion of the tennis sports robot under different classifiers.

**Evaluation parameters**	**SVM classifier**	**Decision tree classifier**	**Random forest classifier**	**Multi-layer perceptron (MLP) classifier**
Accuracy	0.99	0.73	0.99	0.99
Recall	0.99	0.72	0.99	0.98
*f*_1_ score	0.99	0.72	0.99	0.98
Model call time	0.00015	0.00001	0.00002	0.00001

Table 2 shows that the SVM classifier is superior to the decision tree classifier, random classifier, and MLP classifier in action recognition of the tennis sports robot in the accuracy, recall rate, *f*_1_ score, and model call time. It has high accuracy and meets the tennis sports robot's return action recognition requirements.

### Design of Return Strategy Algorithm for Tennis Sports Robot

**Figure 6:**
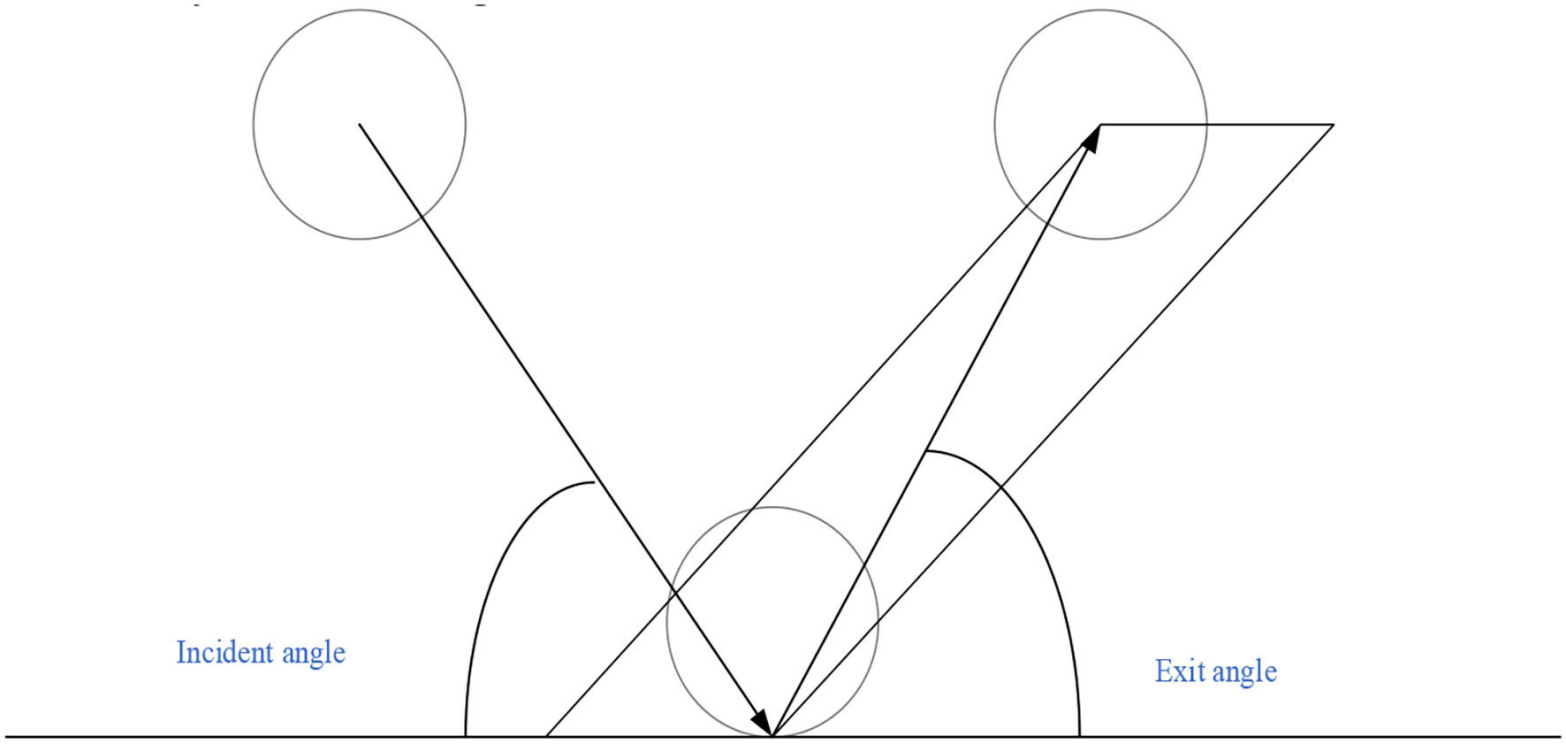
Return state of the tennis sports robot.

The landing areas of the tennis serve are two diagonally opposite areas. Before serving, it is essential to stand in the area behind the end line and between the midpoint and the hypothetical extension line of the sideline, throw the tennis ball into the air, hit the ball with the racket before the ball touches the ground, and complete the sending of the ball when the racket contacts the ball. When facing a tennis player serving, the tennis sports robot cannot change the position of the original station by walking or running. It is best to stand in the specified position and not touch other areas. The player serves from the end line of the right area at the beginning of each game and then changes to the left area to serve after they score or lose points. In serving, the ball should cross the net and fall on the opposite service area in the opposite corner, or it can fall on the surrounding line. However, another service is required if the ball touches the net and falls into the opponent's service area during the service, and the tennis sports robot is not ready to return. Figure 6 shows the return state of the tennis sports robot.

**Figure 7:**
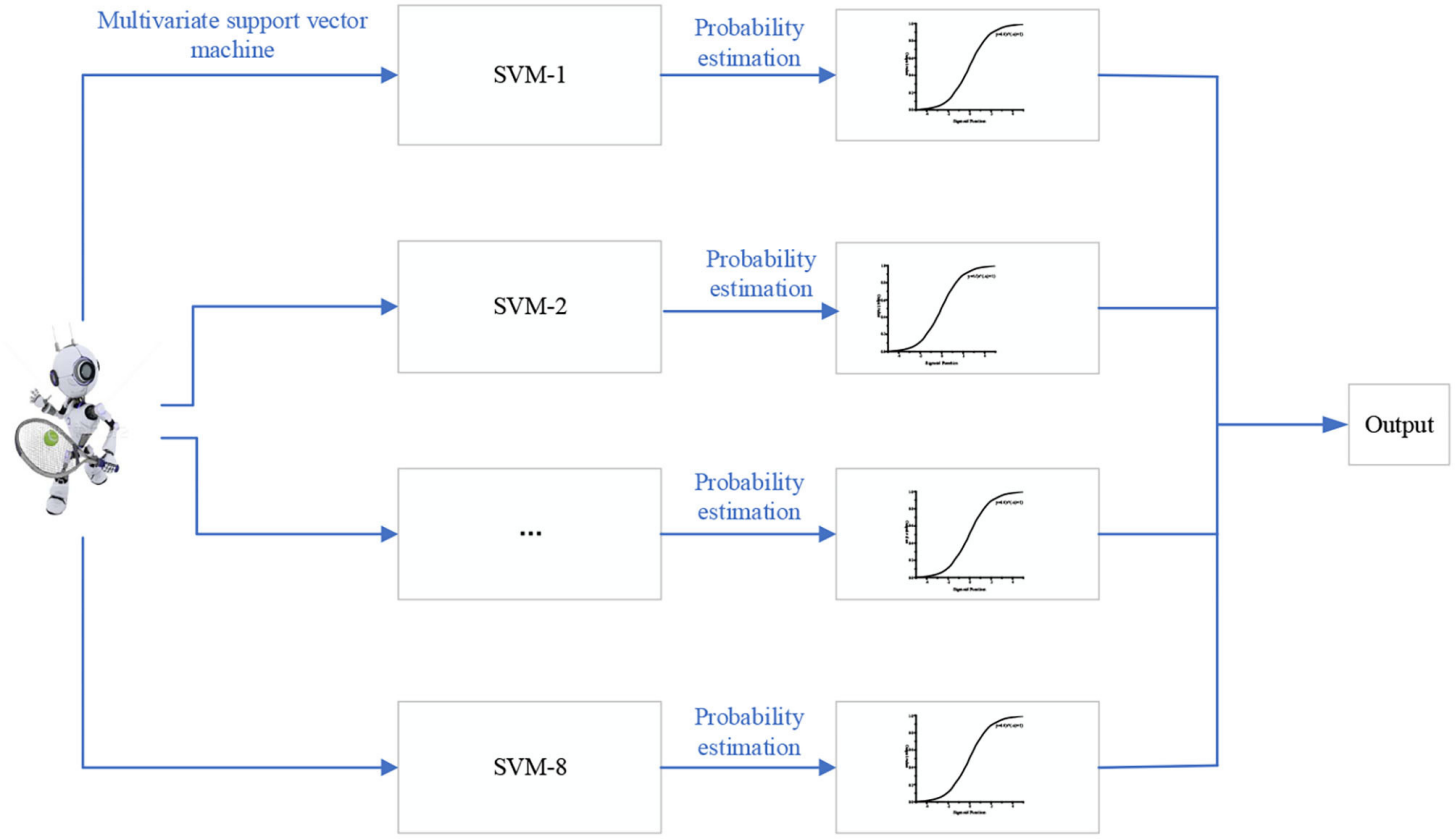
Return strategy model of the tennis sports robot based on SVM.

In Figure 6, after the ball rebounds, the tennis sports robot starts to stare at the ball, which is the same as the ball pressure with a deep landing point. The reason is that the backswing is too late. If the human movement causes the ball speed to reach 260 km/h, the tennis compression is more obvious. The tennis ball forms the incident angle and exit angle with the ground in returning the ball. In a tennis sports robot, the average serving speed of the human body is 150–250 km/h. According to different return points, the opponent serves within a certain speed range no matter how fast the service is. Through the ball return and landing points in different speed ranges, the robot can aim at the other party's ball return and landing point area to return the ball. The tennis sports robot can use SVM to get the optimal return strategy. Figure 7 is the return strategy model of a tennis sports robot based on SVM.

**Figure 8:**
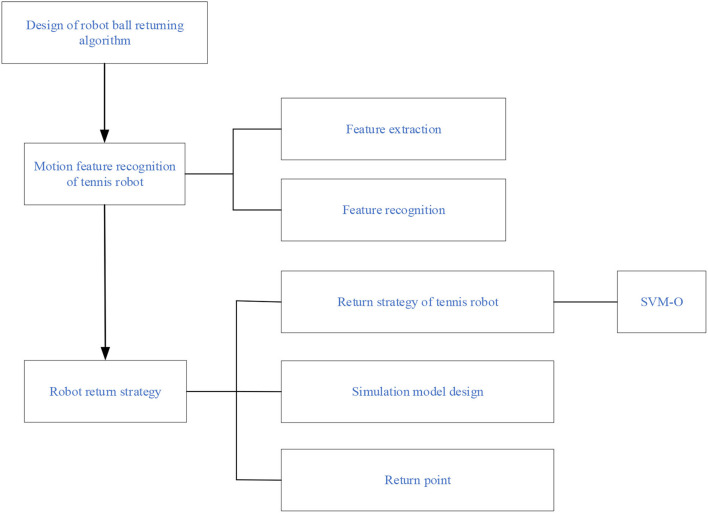
Implementation framework of the return strategy of the tennis sports robot.

Figure 7 is a multivariable SVM learning based on eight ball return actions of a tennis sports robot. However, the maximum interval characterizes the boundary between the current classifier and the dataset because the model is linearly separable from the 2D traditional model. The data volume is modeled and classified based on the feature extraction of human motion recognition images. With the two classifiers in Figures 3, 4 as examples, the maximum interval of blue lines in the curve is greater than that of the black lines. Therefore, the blue line is selected as the classifier. Machine learning is carried out for the return motion of the tennis sports robot, and the probability value is the output through the probability estimation of accuracy. Figure 8 is the implementation framework of the return strategy of the tennis sports robot.

In Figure 8, the algorithm design of the tennis sports robot's return strategy is carried out using machine learning and the SVM-O algorithm. Feature extraction is carried out through the motion characteristics of human motion recognition, and the SVM algorithm is designed and studied for the return ball strategy of the tennis sports robots. Finally, the accuracy of the return strategy of the tennis sports robot is simulated.

## Results and Discussion

### Return Analysis of the Tennis Sports Robot

**Figure 9:**
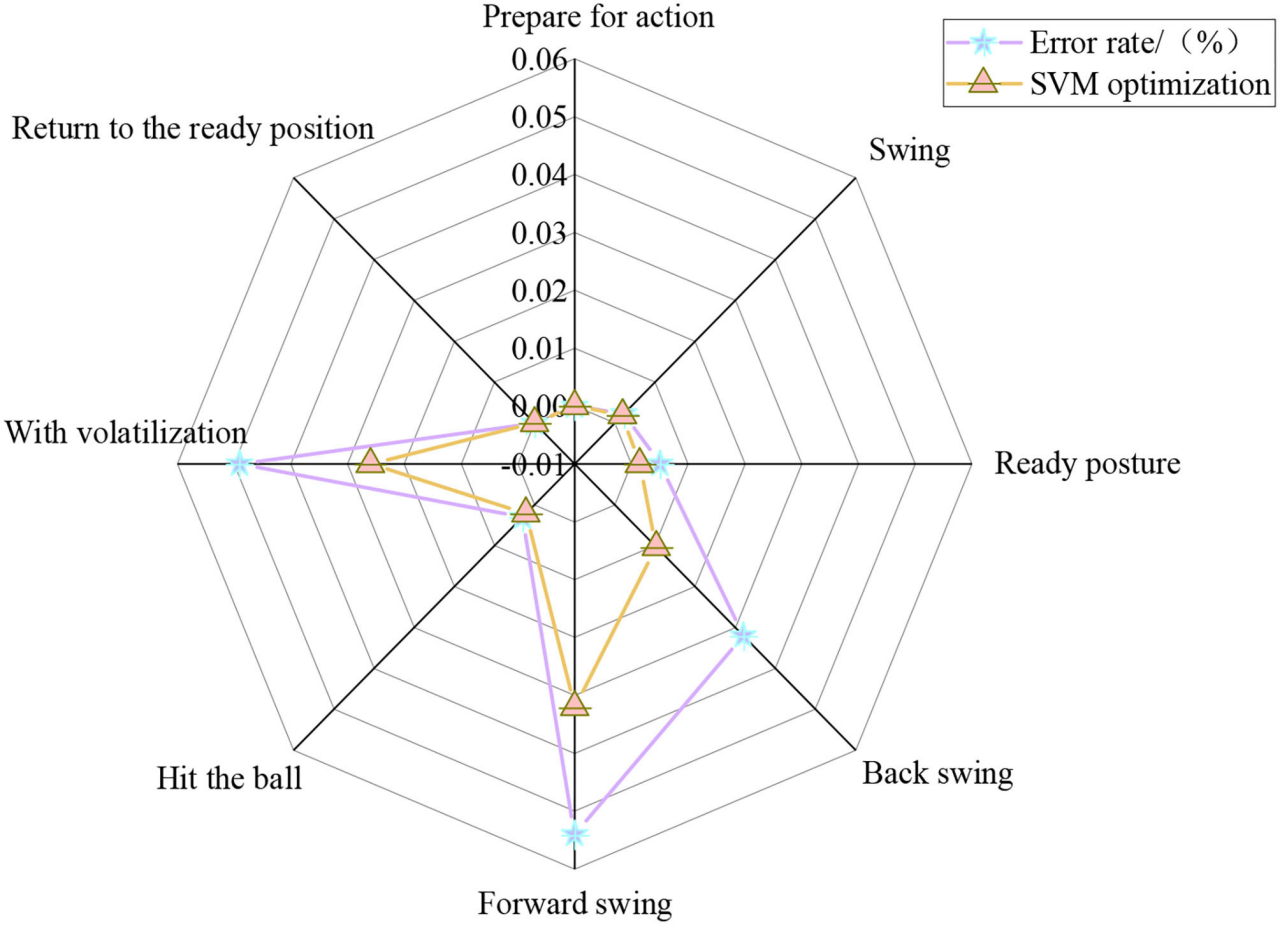
Error rate analysis of 8 kinds of return movements of tennis sports robot based on SVM-O.

To optimize machine learning, the return error rate, return accuracy, return recall rate, return score rate, and return recognition rate of eight actions in the return strategy of a tennis sports robot are analyzed through the optimization analysis of the SVM algorithm. Figure 9 displays the analysis results of the error rate of different return actions of tennis sports robots based on SVM-O.

**Figure 10:**
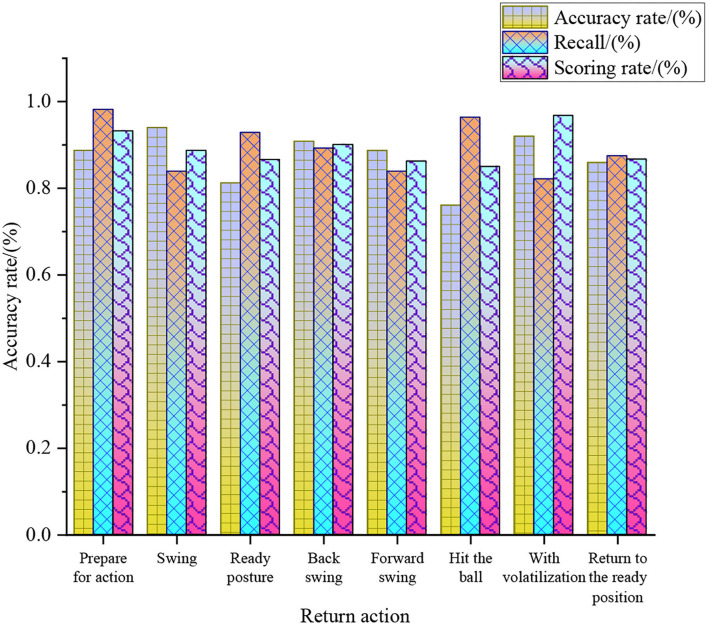
Analysis of different return strategies of the tennis sports robot.

In Figure 9, the average error rates of preparation, swing, ready posture, backswing, forward swing, hitting, volatilization, and returning to the ready position are 0, 0.21, 0.32, 2.12, 4.3, 0.26, 3.75, and 0%, respectively. The error rate of the backswing, forward swing, and volatilization in the return strategy of the tennis sports robot are high. The return accuracy, return recall, and return score of eight return actions are analyzed in varying degrees to further analyze the return strategy of the tennis sports robots. Figure 10 shows the analysis of different return strategies of the tennis sports robots.

**Figure 11:**
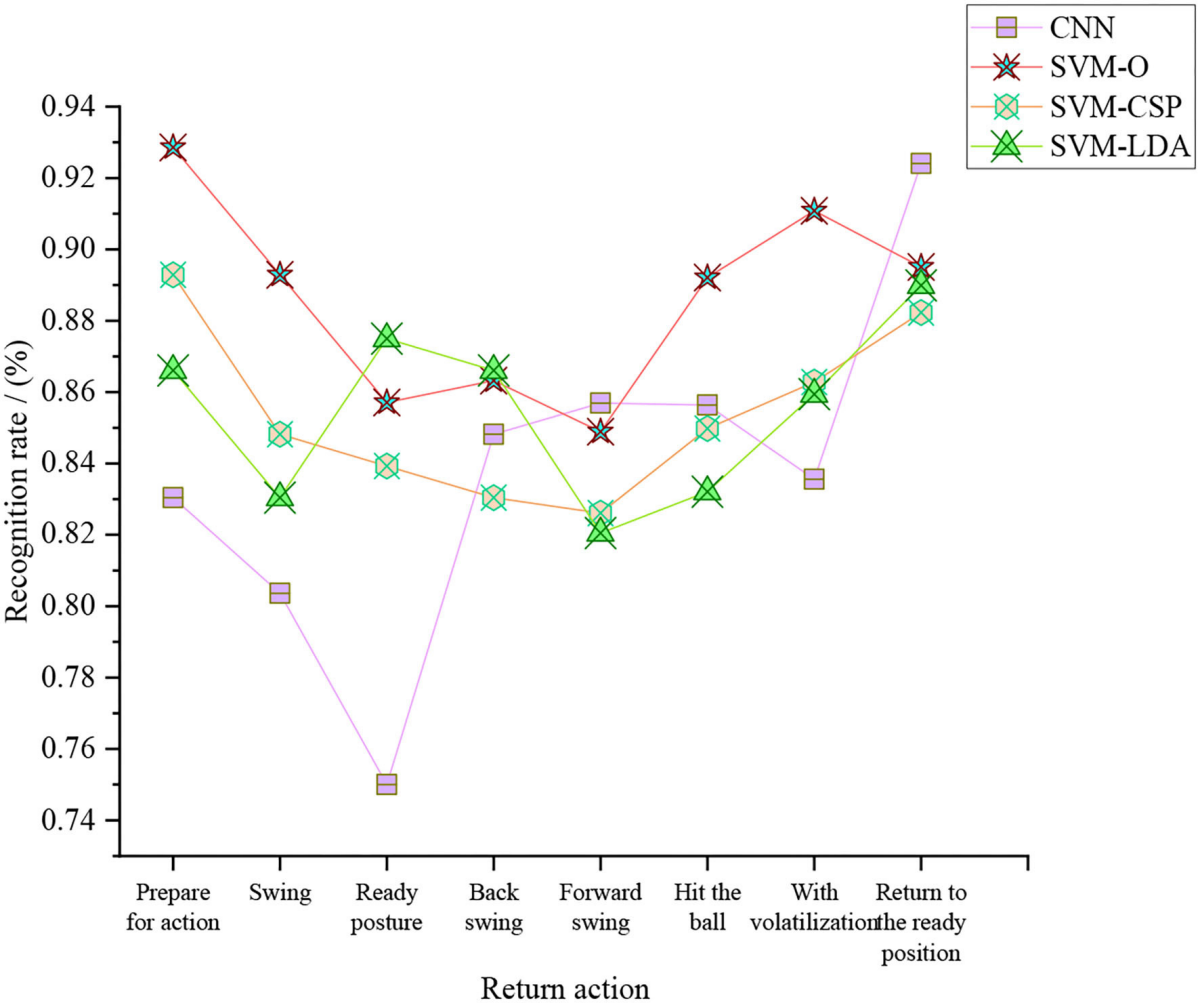
Return recognition rate of the tennis sports robot under different models.

Figure 10 shows that in the ball return strategy of the tennis sports robots after optimization, the action with the highest accuracy rate of machine learning is swing, 94%. The action with the highest recall rate is the preparation action, 98.21%, and the action with the highest-scoring rate is volatilization, 96.79%. Regarding the return accuracy, return recall, and return score, the average values of preparation, swing, ready posture, backswing, forward swing, hitting, volatilization, and returning to the ready position are 93.38, 89, 86.93, 90.10, 86.28, 85.84, 90, and 86.73%. It reveals that in the return strategy of the tennis sports robots, preparation, backswing, and volatilization can achieve more objective results in the analysis of the return strategy. CNN, SVM-Linear Discriminant Analysis (SVM-LDA) algorithm, and SVM—common spatial pattern (SVM-CSP) algorithm are compared and analyzed to study the superiority of the SVM-O algorithm in the ball return recognition rate of the tennis sports robot. Figure 11 shows the return recognition rate of the tennis sports robots under different models.

Figure 11 shows that the tennis sports robot based on the SVM-O algorithm has the highest return recognition rate, with an average return recognition rate of 88.61%. Then, the average return recognition rates of the tennis sports robot based on the SVM-LDA algorithm, SVM-CSP algorithm, and CNN algorithm are 85.49, 85.49, and 83.82%.

### Simulation Effect of the Return Accuracy of the Tennis Sports Robot Under Different Model Training

**Figure 12:**
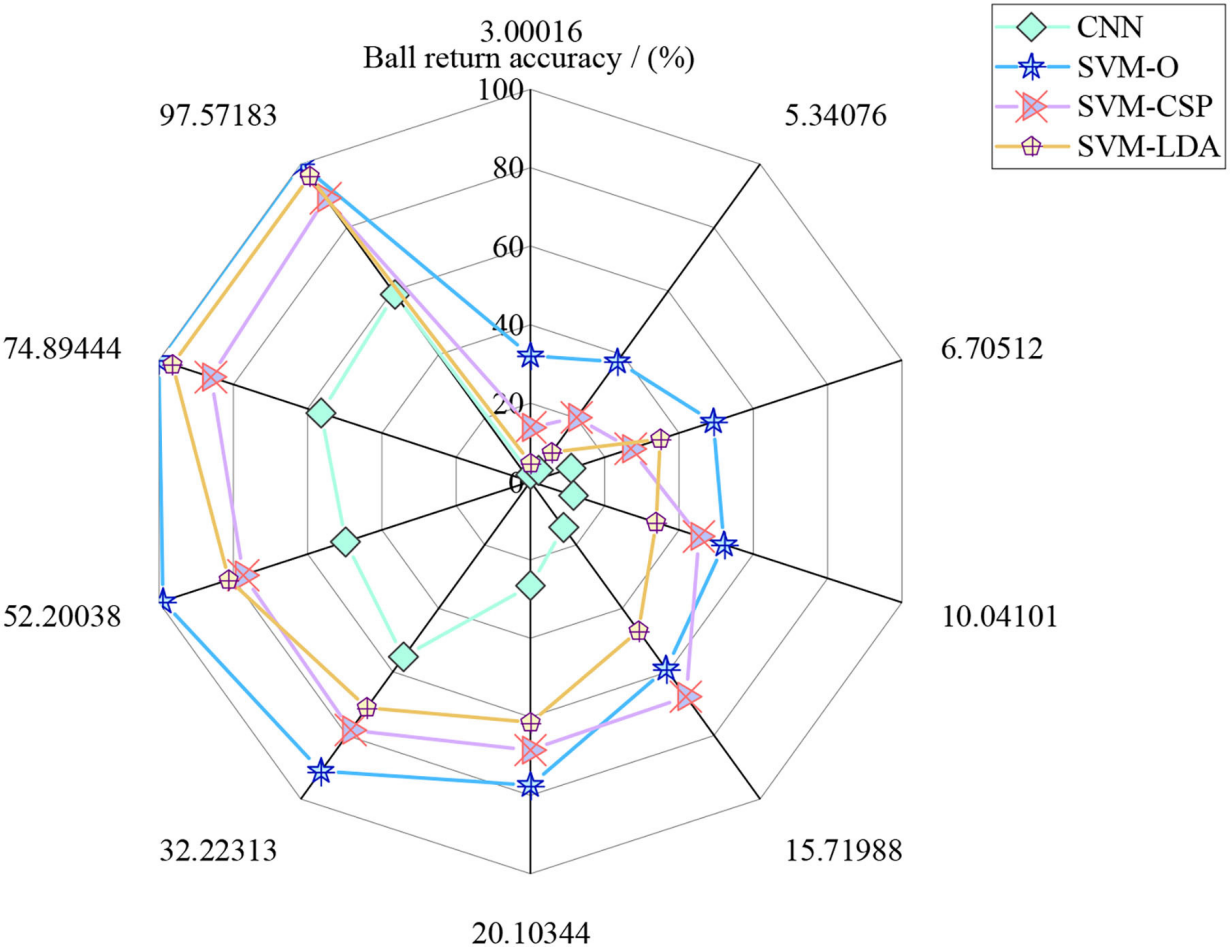
Simulation effect of the return accuracy of the tennis sports robot.

The maximum number of iterations is set to 1 million times in the whole robot return strategy to refer to the final accuracy of the tennis sports robot return. In the simulation process, different models are simulated with different iteration times to simulate the return accuracy of the tennis robot. Figure 12 is the simulation effect of the return accuracy of the tennis sports robot.

Figure 12 shows that when the base number of simulation times is small, there is little difference between the four models for the return accuracy of the tennis robot. With the increase of the iteration times, the effect of the model simulation experiment based on SVM-O is the best, followed by SVM-LDA, SVM-CSP, and finally CNN.

### Discussion

First, the OpenPose traversal dataset was used to extract the information features of the human motion recognition nodes. Then, 8 kinds of tennis robots' return movements were integrated with the human motion characteristics, and the 8 kinds of tennis robots were used to analyze the return movements. By selecting these 8 basic return movements, the complexity of the human motion recognition process in tennis sports robots is reduced. The results show that when the number of return actions of the robot is set to 1 million, the recognition accuracy of the tennis sports robot to the CNN model is too small, and it is not easy to distinguish the return actions of the tennis sports robot. The smaller the number of iterations, the greater the impact on the recognition accuracy. The automatic ball-picking robot operates on the image data according to the previous tennis movement. The eagle eye system is composed of multiple high-speed cameras. Through the application of image recognition, image fusion, and 3D reconstruction technology, the configuration of the tennis robot is too high, and the return action of the tennis robot is not fully realized. The exploration is done to analyze the return action of the tennis sports robots based on the hitting strength and predicted landing point of the tennis sports robot. It is not easy to distinguish the characteristics of human motion by recognizing and analyzing human motion through the image obtained by the camera. However, the recognition of these feature points is significant. The image acquisition of the robot camera improves the efficiency of computational image recognition. However, when these human action feature points greatly impact the recognition results, this method is not desirable. Therefore, the return strategy of tennis robots is studied and analyzed by combining human motion recognition and robot return action.

## Conclusion

Research and analysis show that there is almost no error rate in the preparation and return to the ready position of return strategy of the tennis robot. The error rate of the backswing, forward swing, and volatilization is higher than that of the other movements. Through the optimized SVM, the accuracy of the swing motion is as high as 94.00%. The action with the highest recall rate is preparation, 98.21%. The action with the highest score is swing, 96.79%. Regarding the return accuracy, return recall, and return score, the average values of preparation action, return action, and volatilization action are higher than the other return actions, which are 93.38, 90.10, and 90%, respectively. The research on the return ball recognition rate of the tennis robots under different models shows that the tennis robots' return ball recognition rate based on the SVM-O algorithm is higher than that of the SVM-LDA algorithm, SVM-CSP algorithm, and CNN algorithm. Its average return ball recognition rate is 88.61%. To sum up, the simulation effect of the tennis robot's return strategy model based on SVM-O is the best, improving the diversity of the tennis robot's return strategies.

The required operations such as picking up, putting, and collecting balls in the tennis court are completed through the previous research on the design of the robot arm of the automatic ball-picking robot in tennis sports. The tennis robot's return strategy implementation model based on the SVM-O is proposed. It can integrate human action recognition with the return action of the tennis sports robot. Applying the SVM-O algorithm to the tennis sports robot's return action recognition solves the problem that the optimization algorithm cannot be applied to real-time requirements. It has important research significance for the application of an optimized SVM algorithm in sports action recognition. Tennis robots can combine different return strategies according to the recognition rate and accuracy of the return action. However, according to the work of the tennis robot, its internal structure will delay the operation of the system. Due to the complexity of human motion recognition, the robot cannot quickly launch the ball return according to the ball return strategy. In the follow-up research, more details can be considered to improve the autonomy of the tennis robot.

## Data Availability Statement

The raw data supporting the conclusions of this article will be made available by the authors, without undue reservation.

## Ethics Statement

Written informed consent was obtained from the individual(s) for the publication of any potentially identifiable images or data included in this article.

## Author Contributions

All authors listed have made a substantial, direct, and intellectual contribution to the work and approved it for publication.

## Funding

This work was supported by the Special Project “Research on the Development and Application of Physical Education in Colleges and Universities” of China Higher Education Society, Research on the influence of the evolution of national student physical health standard on the reform of physical education in colleges and universities (Grant No.: 21TYYB12) and Jiangxi Provincial Sports Bureau Sports Research Project, “Health China” led by the scientific theoretical basis and promotion of national fitness exercise and dance research (Grant No.: 202142).

## Conflict of Interest

The authors declare that the research was conducted in the absence of any commercial or financial relationships that could be construed as a potential conflict of interest.

## Publisher's Note

All claims expressed in this article are solely those of the authors and do not necessarily represent those of their affiliated organizations, or those of the publisher, the editors and the reviewers. Any product that may be evaluated in this article, or claim that may be made by its manufacturer, is not guaranteed or endorsed by the publisher.
